# Activity-Dependent Neuroplastic Changes in Autonomic Circuitry Modulating Cardiovascular Control: The Essential Role of Baroreceptors and Chemoreceptors Signaling

**DOI:** 10.3389/fphys.2020.00309

**Published:** 2020-04-09

**Authors:** Carla Rocha-Santos, Douglas Costa Braga, Alexandre Ceroni, Lisete C. Michelini

**Affiliations:** Department of Physiology and Biophysics, Institute of Biomedical Sciences, University of São Paulo, São Paulo, Brazil

**Keywords:** brainstem, dopamine β-hydroxylase, exercise, hypothalamus, oxytocin, sinoaortic denervation

## Abstract

Aerobic exercise training improves the autonomic control of the circulation. Emerging evidence has shown that exercise induces neuroplastic adaptive changes in preautonomic circuitry controlling sympathetic/parasympathetic outflow to heart and vessels. The mechanisms underlying neuronal plasticity are, however, incompletely understood. Knowing that sinoaortic denervation blocks training-induced cardiovascular benefits, we investigate whether baroreceptors’ and chemoreceptors’ signaling are able to drive neuronal plasticity within medullary and supramedullary pathways controlling autonomic outflow. Male Wistar rats submitted to sinoaortic denervation (SAD) or dopamine β-hydroxylase-saporin lesion (DBHx) and respective controls (SHAM) were allocated to training (T) or sedentary (S) protocols for 8 weeks. After hemodynamic measurements at rest, rats were deeply anesthetized for brain harvesting. The density of DBH and oxytocin (OT) cell bodies and terminals were analyzed in brainstem and hypothalamic brain areas (double immunofluorescence reactions, optic and confocal microscopy). In SHAM rats training augmented the density of DBH+ neurons in the nucleus of solitary tract, increased the density of ascending NORergic projections and the number of DBH+ boutons contacting preautonomic OT+ neurons into paraventricular hypothalamic preautonomic nuclei, augmented the density of local OTergic neurons and enhanced the density of OT+ terminals targeting brainstem autonomic areas. These plastic changes occurred simultaneously with reduced sympathetic/increased parasympathetic activity, augmented baroreflex sensitivity and reduced resting heart rate. SAD reduced the density of both DBH+ fibers ascending from brainstem to paraventricular nucleus of hypothalamus and preautonomic OT+ neurons projecting to the brainstem, abrogated training-induced plastic changes and autonomic adaptive responses without changing the treadmill performance. Minor neuroplastic changes with preserved baroreflex sensitivity were observed in trained rats after partial selective disruption of ascending NORergic projections. Our data indicated that afferent inputs conveyed by arterial baroreceptors and chemoreceptors are the main stimuli to drive both inactivity-induced and activity-dependent neuroplasticity within the autonomic circuitry.

## Introduction

The traditional concept of brain plasticity limited to critical periods during development has been challenged in recent years since it has been recognized that activity dependent brain remodeling also occurs in the adult brain (Pascual-Leone et al., 2005). Indeed, in the last two decades several studies provide evidence of exercise-induced neuroanatomical plasticity in mature/older brain including neurogenesis, dendritic morphological remodeling, synaptic plasticity and angiogenesis within the hippocampus and cortical areas, resulting in improved spatial learning and memory (Colcombe et al., 2004; Pascual-Leone et al., 2005; van Praag, 2008; Hötting and Röder, 2013). Studies from our and other laboratories also showed that physical activity induces remarkable neuroplastic adaptive changes within the preautonomic circuitry controlling sympathetic outflow to heart and vessels (Kramer et al., 2002; Mueller, 2007; Michelini and Stern, 2009; Iellamo et al., 2018).

Analyzing in adult rats the expression/activity of preautonomic neurons within the paraventricular nucleus of hypothalamus (PVN) we observed that exercise training augments gene and protein expression of oxytocin (OT), increases the number of dendritic branches and surface area of OT-positive neurons and the density of their projections to target brainstem areas (Higa et al., 2002; Martins et al., 2005; Higa-Taniguchi et al., 2009; Michelini and Stern, 2009; Cavalleri et al., 2011). Exercise training also increases the neuronal excitability of these preautonomic neurons and improves the vagal control of the heart, thus facilitating both reflex bradycardia and the appearance of resting bradycardia in trained individuals (Higa et al., 2002; Martins et al., 2005; Jackson et al., 2005; Michelini, 2007; Higa-Taniguchi et al., 2009; Cavalleri et al., 2011). These studies confirmed the potentiality of exercise training for inducing neuroplasticity in descending OTergic PVN projections to the dorsovagal complex in the brainstem in response to circulatory demand during exercise, therefore improving the autonomic control of the heart. In a recent paper we also showed that training and aging affect not only the descending OTergic projections but also the ascending noradrenergic (NORergic) pathways from brainstem areas to the autonomic PVN nuclei indicating that exercise alters the plasticity of the entire supramedullary circuitry involved in the modulation of heart control (Santos et al., 2018).

Although exercise-induced cardiovascular responses and neuroplastic adaptive changes in the modulatory circuitry are known, our knowledge on mechanisms underlying neuronal plasticity is, however, limited. It is recognized that beneficial cardiovascular responses to exercise are governed by two main neural mechanisms – the central command (a feedforward control to set the basic pattern of physical activity, in which the PVN is an important hub) and feedback mechanisms driven by peripheral receptors (Mitchell, 1990; Rowell and O’Leary, 1990; Michelini et al., 2015). Previous experimental evidence supports two propositions: (i) the feedforward and feedback mechanisms are linked and operate together to orchestrate cardiovascular adjustments to exercise (Michelini and Stern, 2009; Michelini et al., 2015), (ii) the peripheral information conveyed by arterial baroreceptors and chemoreceptors is crucial for training-induced cardiovascular responses since their removal abrogates all functional benefits (Ceroni et al., 2009). Based on these findings, we hypothesized that peripheral afferents are important stimuli to drive plastic changes induced by exercise training in both medullary and supramedullary circuitries integrating the autonomic control of heart and vessels. Therefore, in the present study we investigated in normotensive rats the combined effects of baroreceptors/chemoreceptors absence and exercise training: (1) on the plasticity of NORergic and OTergic pathways within the hypothalamus and brainstem, (2) on cardiovascular parameters, baroreflex sensitivity and autonomic modulation in each experimental situation. To further validate these observations, plastic changes and functional responses to exercise were also analyzed in other groups of rats with intact baroreceptors and chemoreceptors, but with lesion of ascending NORergic projections from brainstem to the PVN.

## Materials and Methods

### Animals and Ethical Approval

All surgical procedures and experimental protocols were reviewed and approved by the Institutional Animal Care and Use Committee of the University of São Paulo, in compliance with the Ethical Principles in Animal Research of the Brazilian College of Animal Experimentation.

Male Wistar rats aged 2 months were housed on a 12:12-h light-dark cycle and allowed free access to food and water. During 2-weeks adaptation period, rats were preselected for their ability to walk/run on a treadmill (Inbramed, KT-300, 10 sessions, 10–15 min/day, 0.3–0.7 km/h, 0% grade). Only active rats were used in this study. Prior to surgery and during experimental protocols rats were submitted to progressive maximal exercise tests on the treadmill (MET = graded exercise, starting at 0.3 km/h with increments of 0.3 km/h every 3 min up to exhaustion, a valuable index to determine the maximal individual aerobic capacities and to quantify the effects of training and sedentary protocols (Coimbra et al., 2008; Buttler et al., 2017).

### Surgeries

#### Protocol I: Sinoaortic Denervation

Male Wistar rats were anesthetized with ketamine (80 mg/kg *ip*, Fort Dodge Animal Health, Fort Dodge, IA, United States) plus xylazine (12 mg/kg *ip*, Alcon, Fort Worth TX, United States) and submitted to SAD as described by Ceroni et al. (2009). Briefly, the neurovascular trunk was exposed bilaterally in the neck. In half of rats the common carotid artery, the vagus and the sympathetic nerves were dissected to allow the identification and specific sectioning of aortic depressor nerves (traveling together the sympathetic nerves and/or as a separate branch) with preservation of the sympathetic trunk. A third contingent of aortic afferent fibers travels along the inferior laryngeal nerve and was interrupted by resection at its junction with the superior laryngeal nerve. The carotid bifurcation was exposed bilaterally for resection of the sinus and carotid body nerves. The other half of rats (SHAM group) was subjected to the same procedure, except by the sectioning of aortic depressor nerves and resection of sinus and carotid body nerves. Rats were then treated subcutaneously with analgesic (ketoprofen 1%, 2 mg/kg, Biofarm, Jaboticabal, SP, Brazil) and penicillin (24,000 IU/kg, Pentabiotico Veterinario, Fontoura Wyeth, Brazil) given subcutaneously and allowed to recover for 7 days.

#### Protocol II: Immunotoxin Lesion (DBHx)

Rats were anesthetized with ketamine plus xylazine and placed in a stereotaxic apparatus (Kopf Instrument, 1760, Tujunga, CA, United States) in the flat-skull position. Bilateral microinjections (0.3 mm needle injector coupled to a 5 μl Hamilton syringe) of the toxin anti-dopamine beta-hydroxylase-saporin (21 ng/100 nL; anti-DβH-SAP, Advanced Targeting Systems, San Diego, CA, United States) or saline were made into the PVN using the following coordinates: 1.8 or 2.0 mm caudal to Bregma, ±0.4 or 0.6 mm lateral to the midline and 7.2 or 7.0 mm below the dura mater, directed to ventromedial or posterior PVN nuclei, respectively, important areas for autonomic control. At the end of the surgery, rats were treated subcutaneously with analgesic and penicillin and recovery for 7 days in their home cages at room temperature (Silva et al., 2016; Bienkowski and Rinaman, 2008).

### Exercise Training and Sedentary Protocols

After surgeries rats were readapted to the treadmill for 1 week and then submitted to a 2nd MET to evaluate the effects of surgery and determine the training intensity. The results of 2nd MET were also used to allocate rats with equal treadmill performance to the training (*T* = 50–60% of maximal exercise capacity, 5 days/week, 1 h/day) and sedentary protocols (S) for 8 weeks. METs were repeated at the 4 and 8th week to correct the exercise intensity and compare the efficacy of T and S protocols among groups, respectively (Dufloth et al., 1997; Braga et al., 2000). Rats assigned to the S groups were handled every day and performed once a week a 10-min exercise in the treadmill.

### Functional Measurements

At the end of the T and S protocols, one day before the functional experiments rats were anesthetized with ketamine + xylazine for catheterization of the femoral artery and vein. Arterial pressure (AP) and heart rate (HR) were measured 24 h after catheters’ implantation and 26–30 h after the last training session in conscious freely moving rats resting in their home cages. The arterial catheter was connected to the recording system (PowerLab, ADInstruments, NSW, Australia, 2000 Hz of sampling frequency), and a variable period of time (20–30 min) was allowed for stabilization of cardiovascular parameters (Amaral and Michelini, 1997) before starting the simultaneous measurement of AP and HR for ∼40 min (basal values). The baroreceptor reflex control of HR (loading/unloading of baroreceptors with phenylephrine and sodium nitroprusside *iv*) was tested to determine lesion effects on baroreflex sensitivity and confirm the SAD procedure (Ceroni et al., 2009; Cavalleri et al., 2011). Time series of systolic AP (SAP) and pulse interval (PI) obtained were also used to evaluate pressure and HR variabilities at the frequency domain, as described before (Santos et al., 2018). Power spectra density at the *very low-frequency* (VLF, <0.20 Hz, reflecting the hormonal modulation of cardiovascular parameters), *low-frequency* (LF, 0.2-0.75 Hz, indicating mainly the sympathetic activity to vessels and sympathetic + parasympathetic modulation of the heart) and *high-frequency* bands (HF, 0.75–3.0 Hz, an index of parasympathetic activity to the heart) were obtained using fast Fourier transformation by Welch’s method and Hanning windows with 50% overlap plus a customized routine (MATLAB 6.0, Mathworks, Natick, MA, United States).

### Tissue Collection and Immunohistochemical Studies

After functional measurements, rats were deeply anesthetized by a bolus of ketamine + xylazine (300 mg/kg + 60 mg/kg, *ip*) and immediately after the respiratory arrest, submitted to transcardiac perfusion with 0.01 Dulbecco’s Modified Eagle’s Medium (300 ml of DMEM, D-8900, Sigma-Aldrich, MS, United States; Daigger Pump, Vernon Hills, IL, United States) followed by fixative (4% paraformaldehyde in 0.1 M PBS, pH 7.2, 500 ml). Rats were decapitated, the brains were removed, postfixed (4% paraformaldehyde for 48 h) and cryoprotected (0.1 M Tris–PBS containing 20% sucrose for a minimum of 24 h followed for 0.1 M Tris–PBS containing 30% sucrose) at room temperature and then stored at 4°C until processing. Coronal sections (30 μm, Leica Cryostat CM3050) from the hypothalamus (1.7 up to 2.2 mm caudal to Bregma) and brainstem (NTS: 13.68 up to 14.40 mm caudal to Bregma; RVLM/C1: 12.24 up to 12.48 mm caudal to Bregma; CVLM/A1: 13.68 up to 14.40 mm caudal to Bregma, (Paxinos and Watson, 2009) were collected in tissue culture wells with 0.01 M PBS at 4°C. Sections were incubated with 0.01% Triton X-100 and 10% normal donkey serum for 30 min. Double immunofluorescence reactions was made as previously described (Santos et al., 2018). Briefly, sections suspended in Tris–PBS containing 0.01% Triton X-100 and 1% normal donkey serum were incubated with a mixture of primary antibodies [polyclonal guinea pig anti-oxytocin (Bachem, Torrance, CA, United States, 1:200,000 dilution) + monoclonal mouse anti-DBH (Millipore, Temecula CA, United States, 1:3,000 dilution) for 24 h], followed by 5-h incubation with secondary antibodies [donkey anti-guinea pig Alexa 594 labeled (1:500 dilution) + donkey anti-mouse Alexa 488 labeled (1:500 dilution), Jackson Immuno Research Laboratories]. Control experiments omitted the primary or the secondary antibodies. Antibodies specificity was confirmed previously (Santos et al., 2018).

### Immunofluorescence Analysis

The histological sections were carefully examined (Zeiss, Wetzlar, Germany) to localize the hypothalamic and brainstem nuclei. The images from the medial and posterior PVN and medullary areas [caudal and intermediate NTS, DMV, CVLM (A1 neurons) and RVLM (C1 neurons)] from all experimental groups were digitized with identical acquisition settings and analyzed. Immunoreactive signals were acquired and quantified as previously described (Higa-Taniguchi et al., 2007; Higa-Taniguchi et al., 2009; Santos et al., 2018). Background intensity was calculated from random adjacent areas of labeled neurons or fibers. The threshold was set to pass intensities 1.5-fold above background immunofluorescence for OTergic neurons (PVN), 2.0-fold for DBH-positive neurons (brainstem areas) and 3.0-fold for DBH (PVN) and OT (brainstem) fibers (Santos et al., 2018). Imaging analysis was performed with ImageJ software (NIH, Bethesta, MD, United States). Regions of interest (ROIs) of predetermined sizes were drawn bilaterally within the following PVN subnuclei (posterior, *post*; ventromedial, *vm*; lateral magnocellular, *mg*) and brainstem areas (NTS, DMV, CVLM, and RVLM). OT and DBH integrated densities (expressed as the product of percent area occupied by the thresholded signal/total ROI area × the signal intensity) were calculated in several slices and averaged to obtain the mean value for each nucleus per rat. Mean integrated density values were then obtained for each experimental condition in each group.

### Confocal Analysis

Selected PVN slices from sedentary and trained SAD and respective controls were reexamined with a Zeiss LSM 780-NLO confocal microscope (Wetzlar, Germany) of the Core Facilities of Biomedical Science Institute, University of São Paulo. Argon (488 nm) and HeNe (543 nm) lasers were used to excite the Alexa 488 and Alexa 594 fluorochromes. Images were digitized with identical microscope settings. Stacks of confocal images containing the two channels and spanning the whole extension of double-labeled neurons (∼50 sections, *z* step: 0,5 μm, image size 1,024 × 1,024 pixels, × 63 oil immersion lens) were obtained from two PVN rostrocaudal levels (1.72–1.80 mm and 2.04–2.16 mm caudal to the Bregma, interesting the PVN*vm* and PVN*post*, respectively). All double-labeled neurons whose somata were fully contained within the *z* stack were selected for quantification (Santos et al., 2018). The image stacks were imported into Imaris software (Bitplane, Oxford Instruments, Switzerland), whose algorithms enabled the 3-dimentional reconstruction of the OTergic neuron and colocalization analysis, discriminating between DBH-positive boutons that overlap the OT neuron from those that do not. The density of overlapping boutons was calculated and expressed as DBH+ boutons/OT+ neuron (videos in Supplementary Material illustrate the analysis made). Since the enzyme DBH converts dopamine into norepinephrine, the presence of DBH immunoreactivity indicates that DBH+ fibers (and neurons) are noradrenergic.

Some PVN images of the DBHx group and respective controls were also selected and reanalyzed by confocal microscopy in order to confirm the effect of saporin lesion on DBH+ terminals projecting to PVN OTergic neurons.

### Statistical Analysis

Data are submitted to Levene’s test for homogeneity of variance and expressed as means ± SEM. The treadmill performance during T and S protocols was analyzed by two-way ANOVA with repeated measurements (time). The effects of exercise (training vs. sedentary) and surgery (DBHx or SAD vs. SHAM) on hemodynamic parameters, OT and DBH immunoreactivity within the PVN nuclei and brainstem areas were analyzed by two-way factorial ANOVA. Bonferroni was used as the *post hoc* test. All analyzes were performed using STATISTICA 12.0 (Vince Stat Software Inc., Palo Alto, CA, United States). Differences were considered significant at *P* < 0.05.

## Results

### Effects of Removal of Afferent Baroreceptors and Chemoreceptors’ Signaling

**FIGURE 1:**
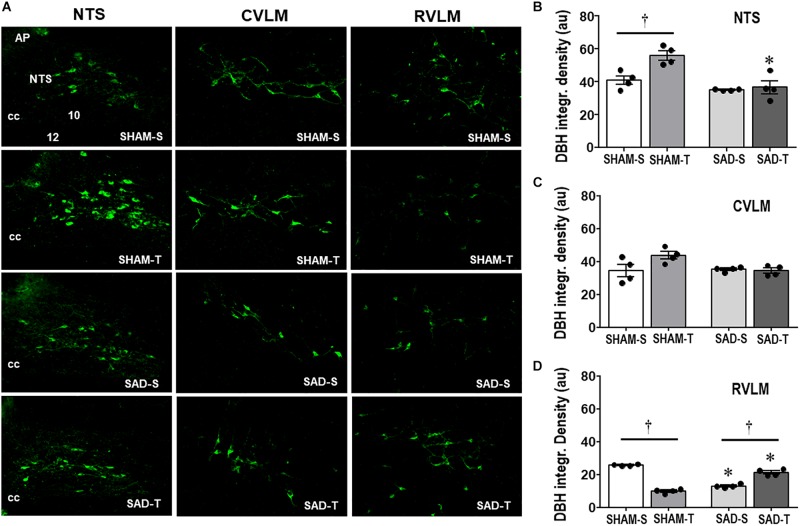
**(A)** Photomicrographs showing changes of DBH + neurons within the NTS, CVLM, and RVLM in sinoaortic denervated (SAD) and sham-operated (SHAM) rats submitted to training (T) or sedentary (S) protocol. AP, area postrema; cc, central canal; 10, DMV; 12, hypoglossus nucleus. Bar graphs depict the quantification of DBH integrated density within the NTS **(B)**, CVLM **(C)**, and RVLM **(D)** in the four experimental groups. Values are the means of 6–9 slices/rat (NTS) and 2–4 slices/rat (CVLM and RVLM), four rats in each subgroup. Comparisons made by Factorial two-way ANOVA. NTS: *group F*(1,12) = 21.62, *P* < 0.001, *condition F* (1,12) = 9.35, *P* = 0.010, *interaction F*(1,12) = 6.15, *P* = 0.029; CVLM: *group F*(1,12) = 3.21, *P* = 0.098, *condition F*(1,12) = 3.19, *P* = 0.100, *interaction F*(1,12) = 4.71, *P* = 0.051; RVLM: *group F*(1,12) = 0.03, *P* = 0.858, *condition F*(1,12) = 28.88, *P* < 0.001, *interaction F*(1,12) = 280.19, *P* < 0.001. Significances (*P* < 0.05) are ° vs. Sedentary and * vs. respective SHAM group.

Knowing the importance of baroreceptors’ and chemoreceptors’s afferent signaling for primary brainstem integration and supramedullary modulation of cardiovascular control (Dampney, 1994; Sved and Gordon, 1994), we evaluated the effects of SAD on those circuitries and on the adaptive responses to treadmill training. Photomicrographs taken from DBH+ neurons within the NTS, CVLM, and RVLM of sedentary and trained SAD and SHAM rats (Figure 1A) illustrated that removal of peripheral afferents did not change the integrated density of DBH+ neurons within the NTS and CVLM (Figures 1B,C), but reduced that of the RVLM (−49%, Figure 1D). SAD also abrogated training-induced augmentation of DBH immunoreactivity observed in the NTS of SHAM-T rats (+37% vs. SHAM-S, Figure 1B) and reversed the effect of training on RVLM DBH integrated density from a reduction in SHAM-T (−61%) to an augmentation in SAD-T group (+62%, Figure 1D) vs. respective S controls. Only a marginal, not significant interaction effect (*P* = 0.051 for group × condition, Figure 1C) was observed in DBH integrated density within the CVLM.

**FIGURE 2:**
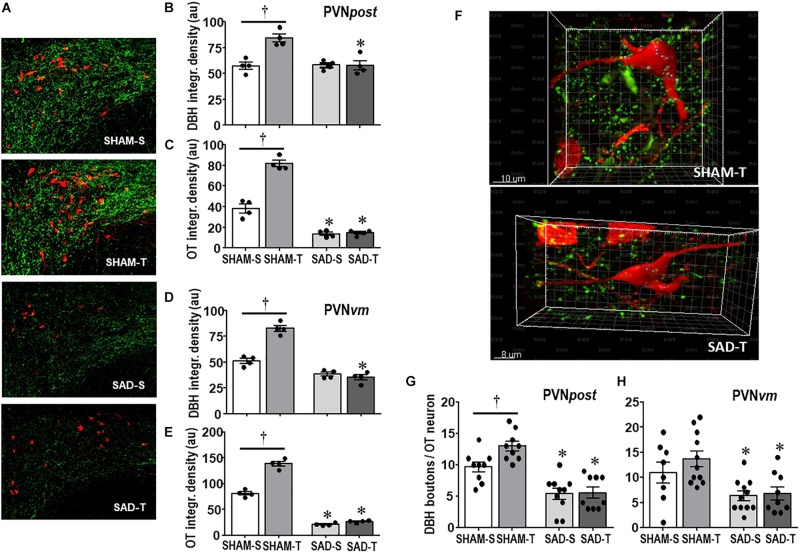
**(A)** Photomicrographs showing changes in DBH + fibers (green) and OT+ neurons (red) within the posterior paraventricular nucleus (PVN*post*) in sinoaortic denervated (SAD) and sham-operated (SHAM) rats submitted to exercise training (T) or sedentary (S) protocol. Bar graphs compare the integrated density of DBH + projections **(B, D)** and OT+ neurons **(C, E)** within the PVNpost **(B, C)** and PVN*vm*, **(D, E)** in the 4 experimental groups. Values are the means of 2–4 slices/rat, four rats in each subgroup. **(F)** Three-dimensional rendered images comparing the density of overlapping boutons (blue) and those that are close, but not overlapping (green) with OT+ neurons (red) taken from PVN*post* of SHAM-T and SAD-T rats. Right bottom graphs compare the effects of SAD and training on the number of DBH + boutons/OT+ neuron within the PVN*post* (**G**) and PVN*vm* (**H**) in the four experimental groups. Values are the means of 6–12 neurons for ROI, 3–4 ROIs/rat, three rats in each subgroup. Comparisons made by Factorial two-way ANOVA. **PVN*post*** – DBH density: *group F*(1,12) = 12.54, *P* = 0.004, *condition F*(1,12) = 13.67, *P* = 0.003, *interaction F*(1,12) = 14.04, *P* = 0.003; OT density: *group F*(1,12) = 106.44, *P* < 0.001, *condition F*(1,12) = 11.74, *P* = 0.005, *interaction F*(1,12) = 5.98, *P* = 0.031. **PVN*vm*** – DBH density: *group F*(1,12) = 142.60, *P* < 0.001, *condition F*(1,12) = 31.56, *P* < 0.001, *interaction F*(1,12) = 47.47, *P* < 0.001; OT density: *group F*(1,12) = 846.68, *P* < 0.001, *condition F*(1,12) = 115.72, *P* < 0.001, *interaction F*(1,12) = 77.47, *P* < 0.001. **DBH + boutons/OT+ neuron** – PVN*post*: *group F*(1,33) = 48.67, *P* < 0.001, *condition F*(1,33) = 4.32, *P* = 0.046, *interaction F*(1,33) = 3.58, *P* = 0.067; PVN*vm*: *group F*(1,35) = 15.14, *P* < 0.001, *condition F*(1,35) = 1.11, *P* = 0.299, *interaction F*(1,35) = 0.60, *P* = 0.442. Significances (*P* < 0.05) are ° vs. Sedentary and * vs. respective SHAM group.

We also analyzed the effects of SAD and training on the expression of ascending DBH projections/terminals to the PVN and local cell bodies of OTergic neurons. In sedentary rats SAD did not change the overall density of DBH+ fibers/terminals in selected ROIs of the PVN*post* (Figures 2A,B) and PVN*vm* (Figure 2D), but it did reduce the density of OT+ neurons within both nuclei (from 38.2 ± 4.2 to 13.7 ± 1.6, and from 80.9 ± 3.3 to 21.0 ± 1.3 arbitrary units into the PVN*pos*t and PVN*vm*, Figures 2C,E, respectively). SAD also abrogated in both nuclei the robust training-induced augmentation on DBH terminals and OT cell bodies immunoreactivity exhibited by SHAM-T vs. SHAM-S rats (DBH = +47%, OT = +2.1-fold, Figures 2B,C and DBH = +62%, OT = +1.7-fold, Figures 2D,E, for PVN*post* and PVN*vm*, respectively). To investigate further the relationship between ascending NORergic fibers/terminals and OTergic neurons within preautonomic PVN areas, we acquired confocal images to analyze the overlap of DBH+ terminals in three-dimensionally reconstructed OT+ neurons. This analysis allowed us to visualize the co-localization of both suggestive of the presence of a synaptic contact and to differentiate between the NORergic projections that actually project to an OT+ neuron from those that only pass through the PVN. Videos of three-dimensionally reconstructed neurons exemplify for each experimental group the results obtained (see Supplementary Figures S1–S4). As shown in the videos, the rotation of the image enabled a precise quantification of DBH+ boutons contacting the cell body and proximal dendrites of a specific reconstructed OT neuron. As observed, removal of baroreceptors’ and chemoreceptors’ afferent signaling reduced the number of overlapping boutons within the PVN*post* (from 9.7 ± 0.8 in SHAM-S to 5.4 ± 0.9 boutons/OT+ neuron in SAD-S, *P* < 0.05, Figure 2G). In SHAM rats exercise training was accompanied by a 34% increase in DBH+ boutons/OT+ neuron and a 2.1-fold augmentation in OTergic density into the PNV*post* (Figures 2G,C, respectively). In contrast, removal of peripheral afferents abrogated these responses as depicted in reconstructed neurons taken from SHAM-T and SAD-T (Figure 2F, quantitative data in Figure 2G), therefore blocking the training-induced plasticity in OT+ neurons. Similar, but mild effects were observed in the PVN*vm* (Figures 2E,H). Notice that within the lateral magnocellular PVN (PVN*mg*, Table 1) SAD also decreased both DBH+ fibers and OT+ neurons immunoreactivity, but training did not change the density of NORergic terminals and did not alter the plasticity of neuroendocrine OT+ neurons indicating these neurons are not directly involved in autonomic modulation of cardiovascular parameters during exercise.

**TABLE 1:** Integrated density (expressed as arbitrary units, AU) of DBH+ fibers and OT+ neurons within the magnocellular PVN nucleus in sedentary (S) and trained (T) rats submitted to sinoaortic denervation (SAD) or DBH-saporin lesion (DBHx) and respective controls (SHAM).

	**SHAM-S**	**SHAM-T**	**SAD-S**	**SAD-T**
	**(*n* = 4)**	**(*n* = 4)**	**(*n* = 4)**	**(*n* = 4)**
***Sinoaortic denervation***				
DBH+ fibers (AU)	46.9 ± 1.3	46.8 ± 2.0	30.1 ± 5.1*	32.1 ± 1.4*
OT+ neurons (AU)	62.3 ± 5.7	72.1 ± 2.5	21.1 ± 4.5*	19.6 ± 1.0*

	**SHAM-S**	**SHAM-T**	**DBHx-S**	**DBHx-T**
	**(*n* = 4)**	**(*n* = 4)**	**(*n* = 4)**	**(*n* = 4)**

***DBH-saporin lesion***				
DBH+ fibers (AU)	40.6 ± 2.6	48.5 ± 4.3	8.6 ± 0.5*	11.4 ± 1.0*
OT+ neurons (AU)	62.0 ± 2.1	79.9 ± 4.9	19.3 ± 2.0*	9.7 ± 4.4*

Values are means ± SEM. Comparisons made by Factorial two-way ANOVA. ***SAD* × *SHAM groups***: DBH+ fibers: *group F*(1,12) = 29.53, *P* < 0.001, *condition F*(1,12) = 0.11, *P* = 0.744, *interaction F*(1,12) = 0.13, *P* = 0.726; OT+ neurons: *group F*(1,12) = 145.59, *P* < 0.001, *condition F*(1,12) = 1.16, *P* = 0.304, *interaction F*(1,12) = 2.13, *P* = 0.170. ***DBHx*** × ***SHAM groups***: DBH+ fibers: *group F*(1,12) = 248.47, *P* < 0.001, *condition F*(1,12) = 4.88, *P* = 0.049, *interaction F*(1,12) = 0.02, *P* = 0.889; OT+ neurons: *group F*(1,12) = 44.53, *P* < 0.001, *condition F*(1,12) = 0.36, *P* = 0.561, *interaction F*(1,12) = 6.12, *P* = 0.029. Significance (*P* < 0.05) is: * vs. respective SHAM group.

**FIGURE 3:**
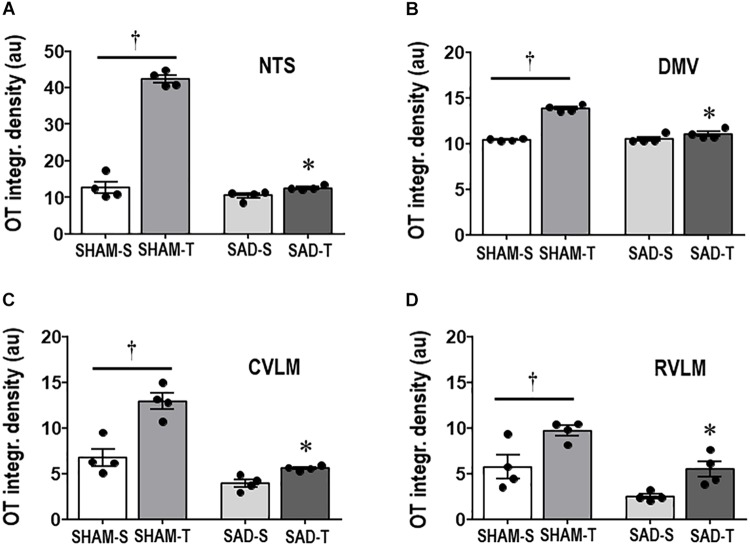
Graphs show the quantification of the integrated density of OT+ projections to the NTS **(A)**, DMV **(B)**, CVLM **(C)**, and RVLM **(D)** in sinoaortic denervated (SAD) and sham-operated (SHAM) rats submitted to training (T) or sedentary (S) protocol. Values are the means of 6–9 slices/rat (DMV and NTS) and 2–4 slices/rat (CVLM and RVLM), four rats in each subgroup. Comparisons made by Factorial two-way ANOVA. NTS: *group F*(1,12) = 236.04, *P* < 0.001, *condition F*(1,12) = 232.33, *P* < 0.001, *interaction F*(1,12) = 175.86, *P* < 0.001; DMV: *group F*(1,12) = 48.10, *P* < 0.001, *condition F*(1,12) = 101.00, *P* < 0.001, *interaction F*(1,12) = 55.81, *P* < 0.001; CVLM: *group F*(1,12) = 556.39, *P* < 0.001, *condition F*(1,12) = 33.07, *P* < 0.001, *interaction F*(1,12) = 10.92, *P* = 0.006; RVLM: *group F*(1,12) = 20.20, *P* < 0.001, *condition F*(1,12) = 17.46, *P* = 0.001, *interaction F*(1,12) = 0.33, *P* = 0.574. Significances (*P* < 0.05) are ° vs. Sedentary and * vs. respective SHAM group.

Next we examined whether SAD- and training-induced adaptive changes in preautonomic OT neurons are accompanied by parallel changes in their projections to primary integrative brainstem nuclei. SAD did not change the integrated density of OT+ terminals within the NTS, DMV, CVLM, and RVLM, but completely abolished in all nuclei the robust increases observed in SHAM-T rats when compared to respective sedentary controls (Figures 3A–D).

In those rats SAD induced a small significant decrease in treadmill performance (average of −0.28 ± 0.06 vs. -0.15 ± 0.04 km/h in SHAM groups, surgery effect in Table 2), but did not interfere with the improvement on treadmill performance observed in trained rats. At the end of protocols gain performance was increased by +48% in SAD-T and +47% in SHAM-T rats, when compared to respective controls at week 0. Gain performance decreased in the SAD-S (−23%, *P* < 0.05, Table 2) while no significant change was observed in the SHAM-S group.

**TABLE 2:** Absolute values of velocity attained during maximal exercise tests before/after surgery, during training (T) and sedentary (S) protocols and performance gain at eight experimental weeks in rats submitted to sinoaortic denervation (SAD) or sham surgery (SHAM). Hemodynamic parameters, baroreflex sensitivity, pressure and HR variabilities and respective spectral components at the end of protocols are also shown.

	**SHAM-S**	**SHAM-T**	**SAD-S**	**SAD-T**
***Treadmill performance***	**(*n* = 14)**	**(*n* = 14)**	**(*n* = 14)**	**(*n* = 14)**
Before surgery (km/h)	1.19±0.06	1.18±0.07	1.20±0.05	1.1±80.05
Surgery effect (km/h)	−0.15±0.04	−0.11±0.04	−0.28±0.06^δ^	−0.25±0.05^δ^
Week 0 (km/h)	1.05±0.08	1.08±0.08	0.92±0.08	0.92±0.05
Week 4 (km/h)	0.86±0.10	1.37±0.07^#⁣†^	0.72±0.07^#^	1.24±0.07^#⁣†^
Week 8 (km/h)	0.96±0.11	1.59±0.08^#⁣†^	0.71±0.07^#^	1.36±0.07^#⁣†^
Gain (w8–w0) (km/h)	−0.09±0.12	+ 0.54±0.07^δ⁣†^	−0.21±0.12^δ^	+ 0.44±0.05^δ⁣†^
***Hemodynamic parameters***	**(*n* = 10)**	**(*n* = 10)**	**(*n* = 9)**	**(*n* = 9)**
MAP (mmHg)	122±3	118±2	131±3*	135±3*
HR (b/min)	378±10	328±8°	364±9	407±15*^†^
***Baroreflex indexes***	**(*n* = 10)**	**(*n* = 10)**	**(*n* = 9)**	**(*n* = 9)**
Bradycardia (bpm/mmHg)	1.77±0.14	2.56±0.23°	0.33±0.10*	0.21±0.06*
Tachycardia (bpm/mmHg)	2.68±0.18	3.59±0.22°	0.48±0.09*	0.31±0.07*
***Power Spectral Analysis***	**(*n* = 10)**	**(*n* = 10)**	**(*n* = 9)**	**(*n* = 9)**
SAP var. (mmHg^2^)	33.3±7.2	14.9±1.7	99.02±3.5*	64.81±9.3*
LF-SAP (mmHg^2^)	7.1±0.7	4.9±0.5	7.7±1.1	6.3±1.1
VLF-SAP (mmHg^2^)	9.1±2.3	4.6±0.7	26.1±9.0*	18.9±3.2*
PI var. (ms^2^)	54.2±6.0	61.5±6.4	29.1±7.6*	37.1±7.6
LF-PI (nu)	44.9±3.3	29.1±2.0	55.1±8.9	40.3±6.0
HF-PI (nu)	52.2±3.5	69.5±2.0	47.1±8.9	53.1±6.4
LF/HF ratio	0.87±0.12	0.43±0.04°	1.68±0.32*	0.82±0.21

Values are means ± SEM. Gain represents the difference in treadmill performance between experimental weeks 0 and 8. Treadmill performance (ANOVA with repeated measurements): *group F*(2,52) = 2.06; *P* = 0.132; *condition F*(2,52) = 17.34, *P* < 0.001, *interaction F*(2,52) = 0.19, *P* = 0.828. Hemodynamic parameters, baroreflex indexes and power spectral parameters (Factorial two-way ANOVA): MAP: *group F*(1,34) = 21.89, *P* < 0.001, *condition F*(1,34) = 0.03, *P* = 0.859; *interaction F*(1,34) = 1.79, *P* = 0.189; HR: *group F*(1,34) = 8.06, *P* = 0.007, *condition F*(1,34) = 0.12, *P* = 0.730; *interaction F*(1,34) = 16.42, *P* < 0.001; Reflex bradycardia: *group F*(1,34) = 161.5, *P* < 0.001, *condition F*(1,34) = 5.05 *P* = 0.031 *interaction F*(1,34) = 9.31 *P* = 0.004; Reflex tachycardia: *group F*(1,34) = 265.2, *P* < 0.001, *condition F*(1,34) = 4.84, *P* = 0.034; *interaction F*(1,34) = 10.30, *P* = 0.003. SAP variability: *group F*(1,34) = 15.98, *P* < 0.001, *condition F*(1,34) = 3.29, *P* = 0.078; *interaction F*(1,34) = 0.30, *P* = 0.588; LF-SAP: *group F*(1,34) = 1.39, *P* = 0.247, *condition F*(1,34) = 4.76, *P* = 0.036; *interaction F*(1,34) = 0.26, *P* = 0.616; VLF-SAP: *group F*(1,34) = 17.95, *P* < 0.001, *condition F*(1,34) = 2.54, *P* = 0.121; *interaction F*(1,34) = 0.13, *P* = 0.718; PI variability: *group F*(1,34) = 12.70, *P* = 0.001, *condition F*(1,34) = 1.20, *P* = 0.283; *interaction F*(1,34) = 0.01, *P* = 0.965; LF-PI: *group F*(1,34) = 3.51, *P* = 0.069, *condition F*(1,34) = 7.18, *P* = 0.011; *interaction F*(1,34) = 0.01, *P* = 0.924; HF-PI: *group F*(1,34) = 3.28, *P* = 0.078, *condition F*(1,34) = 4.61, *P* = 0.039; *interaction F*(1,34) = 0.14, *P* = 0.711; LF/HF: *group F*(1,34) = 5.65, *P* = 0.023, *condition F*(1,34) = 6.63, *P* = 0.014; *interaction F*(1,34) = 0.69, *P* = 0.411; Significant differences (*P* < 0.05) are ^#^ vs. week 0, * vs. Sham, ° vs. Sedentary, **^δ^** means a change different from zero.

Sinoaortic denervation-S vs. SHAM-S (Table 2) exhibited a marked increase in SAP variability (+3.0-fold) augmented VLF-SAP (+2.9-fold) and a slightly higher MAP (+7%, *P* < 0.05). SAD-S also showed reduced PI variability (−46%, *P* < 0.05), altered sympathetic/parasympathetic balance of the heart (LF/HF ratio increased by 1.9-fold), impaired reflex control of the heart (average reduction of 82% in reflex bradycardia and tachycardia) but similar resting HR (Table 2). As expected, the exercise protocol reduced the LF/HF ratio in SHAM rats (−51%), increased both reflex bradycardia and tachycardia (+45 and +34%, respectively), causing the appearance of resting bradycardia (from 378 ± 10 in SHAM-S to 328 ± 8 b/min in SHAM-T), without changing resting MAP. SAD abrogated training effects on both LF/HF ratio and reflex control of the heart, thus reversing the resting bradycardia to tachycardia (407 ± 15 b/min, Table 2).

### Effects of Disruption of the Ascending NORergic Projections to PVN OTergic Neurons

**FIGURE 4:**
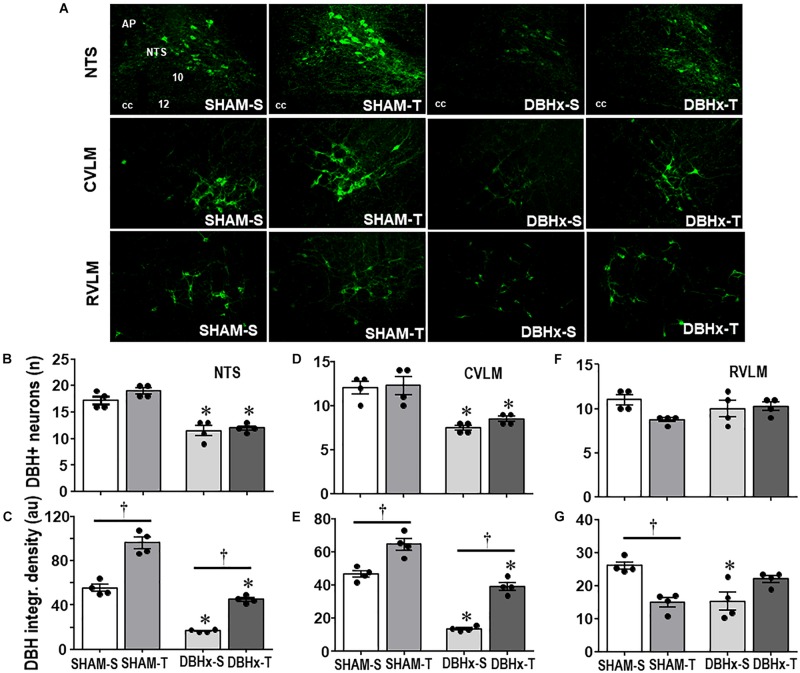
**(A)** Photomicrographs showing changes of DBH + neurons within the NTS, CVLM, and RVLM in rats treated with Dopamine β-hydroxilase-saporin (DBHx) or Saline (SHAM) and submitted to training (T) or sedentary (S) protocol. AP, area postrema; cc, central canal; 10, DMV; 12, hypoglossus nucleus. Bar graphs show the quantification of the number of DBH positive neurons **(B,D,F)** and DBH integrated density **(C,E,G)** in the NTS **(B,C)**, CVLM **(D,E)**, and RVLM **(F,G)** of the four experimental groups. Values are the means of 6–9 slices/rat (NTS) and 2–4 slices/rat (CVLM and RVLM), four rats in each subgroup. Comparisons made by Factorial two-way ANOVA. ***Number of DBH* + *neurons*** – NTS: *group F*(1,12) = 101.10, *P* < 0.001, *condition F*(1,12) = 4.78, *P* = 0.049, *interaction F*(1,12) = 1.30, *P* = 0.277; CVLM: *group F*(1,12) = 36.08, *P* < 0.001, *condition F*(1,12) = 0.97, *P* = 0.345, *interaction F*(1,12) = 0.31, *P* = 0.587; RVLM: *group F*(1,12) = 0.01, *P* = 0.922, *condition F*(1,12) = 3.93, *P* = 0.071, *interaction F*(1,12) = 4.12, *P* = 0.065. ***DBH density*** – NTS: *group F*(1,12) = 293.76, *P* < 0.001, *condition F*(1,12) = 185.40, *P* < 0.001, *interaction F*(1,12) = 8.89, *P* = 0.015; CVLM: *group F*(1,12) = 149.83, *P* < 0.001, *condition F*(1,12) = 83.28, *P* < 0.001, *interaction F*(1,12) = 2.62, *P* = 0.132; RVLM: *group F*(1,12) = 1.13, *P* = 0.309, *condition F*(1,12) = 1.48, *P* = 0.247, *interaction F*(1,12) = 26.08, *P* < 0.001. Significances (*P* < 0.05) are ° vs. Sedentary and * vs. respective SHAM group.

The efficacy of PVN DBH-saporin microinjection in the lesion of ascending NORergic projections to the PVN was evaluated in the cell bodies located in brainstem nuclei and their projection sites within preautonomic PVN areas. Photomicrographs taken from representative rats of each group (Figure 4A) indicated a noticeable reduction in cell bodies positive to DBH staining within the NTS and CVLM of saporin-treated rats, without affecting the number of DBH+ neurons located into the RVLM. Quantitative data confirmed these observations: DBHx-S vs. SHAM-S rats exhibited marked reductions in the number of DBH+ neurons (decreases of −34 and −38% in NTS and CVLM, Figures 4B,D, respectively) that are accompanied by robust reductions in DBH integrated density of −70 and −71% (Figures 4C,E) within the NTS and CVLM nuclei, which are shown to send dense afferent projections to the PVN (Sawchenko and Swanson, 1981). Within the RVLM (the main location of premotor neurons innervating the sympathetic preganglionic neurons projecting to the intermediolateral column in the spinal cord (Swanson and Sawchenko, 1983) saporin lesion did not affect the number of DBH+ neurons, but DBHx-S rats exhibited a slight reduction of DBH integrated density (Figures 4F,G). In all brainstem nuclei training did not change the number of DBH+ neurons (Figures 4B,D,F), but it did increase DBH density within the NTS and CVLM of SHAM rats (+74 and +38%, respectively, Figures 4C,E), while decreasing RVLM DBH immunoreactivity (−43%, Figure 4G). Since DBH-saporin treatment reduced but did not eliminate DBH+ neurons, training-induced augmentation of DBH integrated density was still observed within the NTS and CVLM of DBHx-T group (vs. DBH-S, *P* < 0.05, Figures 4C,E).

**FIGURE 5:**
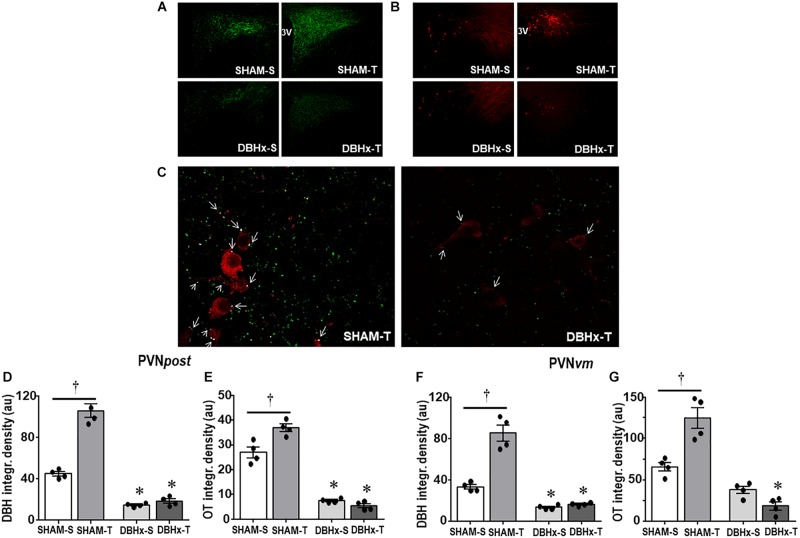
Photomicrographs depicting changes in DBH + fibers (green, **(A)**) and OT+ neurons (red, **(B)**) within the posterior paraventricular nucleus (PVN*post*) in rats treated with Dopamine β-hydroxilase-saporin (DBHx) or Saline (SHAM) and submitted to training (T) or sedentary (S) protocol. 3rd ventricle. **(C)** Three-dimensional rendered images comparing the density of DBH + overlapping boutons (arrows) and those that are close, but not overlapping (green) with oxytocin positive neurons (red) taken from PVN*post* of SHAM-T and DBHx-T rats. Graphs show the quantification of the integrated density of DBH + projections **(D, F)** and OT+ neurons **(E, G)** within PVN*post* **(D, E)** and PVN*vm* **(F, G)** in the 4 experimental groups. Values are the means of 2–4 slices/rat, four rats in each subgroup. Comparison made by Factorial two-way ANOVA. **PVN*post*** – DBH density: *group F*(1,12) = 367.61, *P* < 0.001, *condition F*(1,12) = 52.05, *P* < 0.001, *interaction F*(1,12) = 18.86, *P* < 0.001; OT density: *group F*(1,12) = 322.51, *P* < 0.001; *condition*: *F*(1,12) = 7.68, *P* = 0.017, *interaction*: *F*(1,12) = 17.70, *P* = 0.001; **PVN*vm*** – DBH density: *group F*(1,12) = 257.92, *P* < 0.001, *condition F*(1,12) = 57.90, *P* < 0.001, *interaction F*(1,12) = 17.44, *P* = 0.001; OT density: *group F*(1,12) = 75.41, *P* < 0.001, *condition F*(1,12) = 0.76, *P* = 0.400, *interaction F*(1,12) = 8.49, *P* = 0.013. Significances (*P* < 0.05) are ° vs. Sedentary and * vs. respective SHAM group.

Consistent with the effects observed in brainstem nuclei, DBHx rats showed a pronounced reduction in the density of DBH+ fibers/terminals in selected ROIs within PVN*post* (−67%, Figures 5A,D) and PVN*vm* (−60%, Figure 5F). In both nuclei saporin treatment blocked the marked training-induced augmentation in DBH integrated density observed in SHAM-T (+2.4-fold and +2.6-fold vs. SHAM-S in PVN*post* and PVN*vm*, Figures 5D,F, respectively). Selected images of PVN*post* were also analyzed by confocal microscopy (Figure 5C). To uncover whether saporin lesion also changes the contact of DBH projections with OTergic neurons, we compared the occurrence of DBH boutons overlaying OT+ neurons in DBHx-T and SHAM-T rats and observed a robust reduction after saporin lesion (Figure 5C). Reduction of afferent signaling to OTergic neurons do impact their tonicity and adaptive response to exercise: DBHx-S rats (vs. SHAM-S) showed a significant reduction of OT density within the PVN*post* (−72%, Figures 5B,E) and PVN*vm* (−43%, Figure 5G); coherently, training-induced augmentation of OT immunoreactivity was abrogated in both nuclei after lesion of NORergic ascending fibers to these neurons (Figures 5E,G).

We also measured the density of DBH+ fibers and OT+ neurons within the magnocellular PVN. Saporin also markedly decreased local DBH+ fibers/terminals as well as the immunoreactivity of OT+ neurons (reductions of 79 and 65%, respectively, Table 1). Within these neuroendocrine neurons there was no significant effect of training.

**FIGURE 6:**
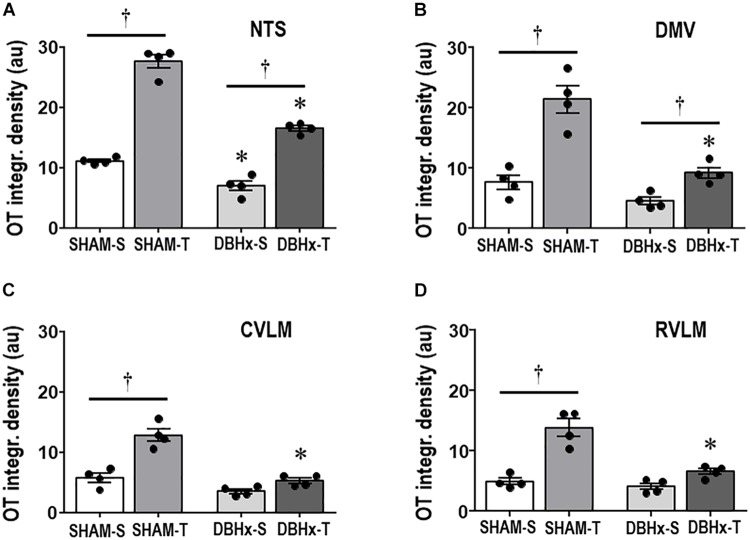
Graphs show the quantification of the integrated density of OT+ projections to the NTS **(A)**, DMV **(B)**, CVLM **(C)**, and RVLM **(D)** in rats treated with Dopamine β-hydroxilase-saporin (DBHx) or Saline (SHAM) and submitted to training (T) or sedentary (S) protocol. Values are the means of 6–9 slices/rat (DMV and NTS) and 2–4 slices/rat (CVLM and RVLM), four rats in each subgroup. OT density comparisons made by Factorial two-way ANOVA. NTS: *group F*(1,12) = 103.51, *P* < 0.001, *condition F*(1,12) = 304.43, *P* < 0.001, *interaction F*(1,12) = 21.81, *P* < 0.001; DMV: *group F*(1,12) = 30.21, *P* < 0.001, *condition F*(1,12) = 44.49, *P* < 0.001, *interaction F*(1,12) = 10.73, *P* = 0.007; CVLM: *group F*(1,12) = 48.15, *P* < 0.001, *condition F*(1,12) = 38.85, *P* < 0.001, *interaction F*(1,12) = 13.93, *P* = 0.003; RVLM: *group F*(1,12) = 18.12, *P* = 0.001, *condition F*(1,12) = 51.22, *P* < 0.001, *interaction F*(1,12) = 9.59, *P* = 0.009. Significances (*P* < 0.05) are ° vs. Sedentary and * vs. respective SHAM group.

Next we analyzed whether saporin- and training-induced plastic changes in preautonomic OT neurons are accompanied by parallel changes in their projections to primary integrative brainstem nuclei. DBHx-S rats exhibited a mild reduction in the density of OT+ terminals within the NTS (−37% vs. SHAM-S, Figure 6A), without significant changes in the other brainstem nuclei. Saporin treatment reduced but did not block training-induced increases in OTergic terminals within the NTS and DMV (+2.4- and +2.0-fold, respectively, Figures 6A,B). Mild, not significant increases in OTergic terminals were also observed in the CVLM and RVLM of DBHx-T vs. DBH-S rats (Figures 6C,D). Similar to the effects observed in OTergic neurons within PVN preautonomic nuclei (DBH+ terminals contacting OT+ neurons and OT immunoreactivity), OTergic projections to all brainstem areas are significantly reduced in DBHx-T when compared to SHAM-T rats.

Rats allocated to these SHAM and DBHx groups exhibited similar treadmill performance at the beginning of the experiments (Table 3). Both surgeries caused a small non-significant reduction on treadmill performance (−0.15 km/h) and did not block the improvement on treadmill performance exhibited by trained groups, which showed a similar gain at the end of protocols (+38%, Table 3). On the other hand, SHAM-S and DBHx-S rats showed a slightly reduced treadmill performance after the eight experimental weeks. At the end of protocols, resting values of MAP, SAP variability, sympathetic vasomotor activity (LF-SAP) and hormonal modulation (VLF-SAP) were similar in both sedentary groups and not changed by training (Table 3). Partial lesion of ascending DBH+ neurons only caused mild not significant changes in the autonomic control of the heart (PI variability, LF-PI and HF-PI values are similar in DBHx-S and SHAM-S). In the SHAM group training increased vagal (+22% in HF-PI) and reduced sympathetic modulation of the heart (−39% in HF-PI), thus augmenting baroreflex sensitivity (average increase of 38%) and reducing the resting HR (−9%, Table 3). Sympathetic and parasympathetic modulation of the heart were not improved in trained saporin-lesioned rats, therefore blocking the appearance of resting bradycardia in DBHx-T rats (Table 3). In contrast, reflex control of the heart activated by loading/unloading the baroreceptors was preserved after partial lesion of ascending NORergic projections, and similarly to SHAM groups, it was significantly improved by aerobic training (Table 3).

**TABLE 3:** Absolute values of velocity attained during maximal exercise tests before/after surgery, during training (T) and sedentary (S) protocols and performance gain at eight experimental weeks in rats microinjected with Dopamine β-hydroxilase-Saporin (DBHx) or saline (SHAM) into the PVN. Hemodynamic parameters, baroreflex sensitivity, pressure and HR variabilities and respective spectral components at the end of protocols are also shown.

	**SHAM-S**	**SHAM-T**	**DBHx-S**	**DBHx-T**
***Treadmill performance***	**(*n* = 7)**	**(*n* = 7)**	**(*n* = 7)**	**(*n* = 7)**
Before surgery (km/h)	1.13±0.14	1.13±0.19	1.13±0.12	1.13±0.14
Surgery effects (km/h)	−0.15±0.09	−0.15±0.15	−0.15±0.09	−0.15±0.09
week 0 (km/h)	0.99±0.19	0.98±0.14	0.98±0.14	0.98±0.13
week 4 (km/h)	0.90±0.12	1.20±0.12	0.83±0.08	1.20±0.12
week 8 (km/h)	0.75±0.09	1.35±0.09^#⁣†^	0.75±0.09	1.35±0.09^#⁣†^
Gain (w_8_–w_0_) (km/h)	−0.23±0.14	+ 0.38±0.07^δ⁣†^	−0.23±0.14	+ 0.38±0.08^δ⁣†^
***Hemodynamic parameters***	**(*n* = 4)**	**(*n* = 4)**	**(*n* = 4)**	**(*n* = 4)**
MAP (mmHg)	113±3	109±4	110±2	109±3
HR (b/min)	352±3	321±4°	351±5	350±3*
***Baroreflex indexes***	**(*n* = 4)**	**(*n* = 4)**	**(*n* = 4)**	**(*n* = 4)**
Bradycardia (bpm/mmHg)	1.81±0.11	2.60±0.07°	1.61±0.09	2.25±0.10°
Tachycardia (bpm/mmHg)	2.47±0.09	3.28±0.16°	2.30±0.14	3.06±0.17°
***Power spectral analysis***	**(*n* = 4)**	**(*n* = 4)**	**(*n* = 4)**	**(*n* = 4)**
SAP variability (mmHg^2^)	17.0±1.3	13.4±0.7	18.0±5.8	16.6±1.4
LF-SAP (mmHg^2^)	3.1±0.4	2.9±0.4	4.1±0.9	2.8±0.3
VLF-SAP (mmHg^2^)	6.9±1.2	6.0±0.4	6.7±1.7	4.3±0.8
PI variability (ms^2^)	32.9±2.1	36.4±0.9	26.9±3.4	33.7±9.1
LF-PI (nu)	29.9±3.2	18.2±2.5°	21.3±0.1	24.1±4.8
HF-PI (nu)	67.6±2.8	82.7±2.7°	78.0±2.5	75.8±5.1
LF/HF ratio	0.47±0.06	0.22±0.04°	0.27±0.04	0.34±0.10

Values are means ± SEM. Gain represents the difference in treadmill performance between experimental weeks 0 and 8. Treadmill performance (ANOVA with repeated measurements): *group F*(2,24) = 0,12; *P* = 0.888; *condition F*(2,24) = 36.12, *P* < 0.001; *interaction F*(2,24) = 0.12, *P* = 0,888. Hemodynamic parameters, baroreflex indexes and power spectral parameters (Factorial two-way ANOVA): MAP: no significant effects for *group, condition* or *interaction*; HR: *group F*(1,12) = 16.96, *P* = 0.001, *condition F*(1,12) = 22.55, *P* < 0.001; *interaction F*(1,12) = 15.74, *P* = 0.002; Reflex bradycardia: *group F*(1,12) = 8.62, *P* = 0.013, *condition F*(1,12) = 58.26, *P* < 0.001, *interaction F*(1,12) = 0.64, *P* = 0.439; Reflex tachycardia: *group F*(1,12) = 1.60, *P* = 0.230, *condition F*(1,12) = 26.56, *P* < 0.001; *interaction F*(1,12) = 0.02, *P* = 0.879. SAP variability: *group F*(1,12) = 1.79, *P* = 0.206, *condition F*(1,12) = 2.21, *P* = 0.163; *interaction F*(1,12) = 0.02, *P* = 0.881; LF-SAP: *group F*(1,12) = 0.52, *P* = 0.485, *condition F*(1,12) = 1.48, *P* = 0.247; *interaction F*(1,12) = 0.71, *P* = 0.417; VLF-SAP: *group F*(1,12) = 0.76, *P* = 0.400, *condition F*(1,12) = 2.11, *P* = 0.172; *interaction F*(1,12) = 0.43, *P* = 0.526; PI variability: *group F*(1,12) = 0.76, *P* = 0.400, *condition F*(1,12) = 1.07, *P* = 0.322; *interaction F*(1,12) = 0.11, *P* = 0.747; LF-PI: *group F*(1,12) = 0.18, *P* = 0.675, *condition F*(1,12) = 2.03, *P* = 0.182; *interaction F*(1,12) = 5.32, *P* = 0.039; HF-*P*I: *group F*(1,12) = 0.26, *P* = 0.620, *condition F*(1,12) = 3.53, *P* = 0.085; *interaction F*(1,12) = 6.34, *P* = 0.027; LF/HF: *group F*(1,12) = 0.41, *P* = 0.534, *condition F*(1,12) = 2.08, *P* = 0.175; *interaction F*(1,12) = 6.56, *P* = 0.025. Significant differences (*P* < 0.05) are ^#^ vs. week 0, * vs. Sham, ° vs. Sedentary, **^δ^** means a change different from zero.

## Discussion

The present set of data confirmed previous observations by us (Ceroni et al., 2009) that sinoaortic denervation abolishes the beneficial adjustments on cardiovascular control induced by low to moderate exercise training. In addition, it shows that baroreceptors’ and chemoreceptors’ activity drive the neuroplastic adaptive changes within medullary and supramedullary modulatory circuitries, since sinoaortic denervation abrogates both neuronal plasticity and improved cardiovascular control in trained rats. Several new observations arise from this study: (i) transient changes in pressure and blood gases during repetitive exercise sessions, by increasing baroreceptors’ and chemoreceptors’ signaling to integrative brain areas, are essential stimuli to augment the immunoreactivity of DBH+ neurons within the NTS and CVLM, the main sources of ascending NORergic projections conveying information to preautonomic PVN nuclei; (ii) increased NORergic drive to posterior and ventromedial PVN augments the number of DBH+ boutons contacting OTergic preautonomic neurons, which exhibit robust plastic changes; (iii) activity-dependent plasticity of OT+ terminals projecting to DMV and RVLM (the origin of preganglionic parasympathetic and sympathetic premotor neurons, respectively) are accompanied by improvement of autonomic control, increased baroreflex sensitivity and HR reduction; (iv) activity-dependent plasticity is also observed in OTergic descending projections from PVN to the NTS and CVLM where sensory NORergic neurons are located, disclosing a prompt feedback circuitry for modulation of afferent ascending information; (v) all these effects are completely abrogated by sinoaortic denervation; and (vi) minor neuroplastic changes in autonomic circuitry with unchanged reflex control of the circulation are observed in trained rats following partial lesion of ascending NORergic projections in the presence of intact afferent signaling, reinforcing the decisive role of baroreceptors’ and chemoreceptors’ activity in driving activity-dependent neuronal plasticity and the consequent functional responses.

It is well established that arterial baroreceptors and chemoreceptors codify changes in blood pressure and blood gases levels into bursts of action potentials, send this sensory information to the NTS and other brainstem areas for neural processing resulting in appropriate efferent responses to maintain cardiovascular and respiratory homeostasis (Dampney, 1994; Sved and Gordon, 1994; Millhorn, 1986). Neuroanatomical studies have shown that peripheral information, via monosynaptic and polysynaptic catecholaminergic projections, ascends from brainstem nuclei to higher brain areas as the hypothalamus, particularly the PVN, an important integrative center for autonomic and neuroendocrine control (Swanson and Sawchenko, 1980; Kalia et al., 1985). It was also shown that OTergic preautonomic PVN neurons project back (via long-descending monosynaptic projections) to primary brainstem integrative areas (Sawchenko and Swanson, 1982; Kalia et al., 1985), providing the anatomical basis for a prompt feedback control loop that modulates primary brainstem integration. This circuitry is able to adjust autonomic responses in different behavioral situations such as the exercise (Higa-Taniguchi et al., 2007; Michelini and Stern, 2009). Indeed, our previous studies in exercised rats showed important adaptive changes in both ascending NORergic afferents from brainstem to the hypothalamus and descending hypothalamic OTergic pathway to the solitary-vagal complex (enhanced NORergic density, augmented expression of PVN OTergic neurons and of OT projections to target areas (Martins et al., 2005; Higa-Taniguchi et al., 2007; Santos et al., 2018) that improved the reflex bradycardia and the slowdown of the heart after exercise training (Higa et al., 2002; Higa-Taniguchi et al., 2009).

The present results, disclosing an important mechanism to trigger neuronal plasticity, extend our knowledge on cardiovascular adaptive responses. By comparing functional parameters and plastic changes in NORergic and OTergic pathways in sedentary and trained rats submitted to sham surgery, we were able to show that repetitive exercise sessions, by increasing the afferent baroreceptor and chemoreceptor signaling, augmented the density of ascending NTS and CVLM DBH+ neurons and the number of terminals contacting preautonomic PVN OT+ neurons, increased the immunoreactivity of these neurons and the density of their projections to brainstem autonomic areas involved in cardiovascular control. These training-induced neuroplastic changes in medullary and supramedullary circuitries were essential to drive the observed functional adjustments as the reduced sympathetic and augmented parasympathetic activity, the improved baroreceptor reflex control and the resting bradycardia. Indeed, a previous patch-clamp study recording the activity of retrograde labeled preautonomic neurons showed that training increased the amplitude and rising time of action potentials and enhanced the input-output function of PVN neurons projecting to the solitary-vagal complex therefore confirming the training-induced functional adjustments (Jackson et al., 2005). In contrast, chronic sinoaortic denervation did not disrupt the ascending noradrenergic pathway from brainstem to PVN, but the chronic absence of afferent information determined important reductions in the number of DBH+ boutons contacting OTergic neurons into the posterior and ventromedial PVN nuclei and markedly reduced the immunoreactivity of OT+ preautonomic neurons projecting to the brainstem. SAD completely abrogated both training-induced neuroplastic changes in the whole autonomic circuitry and cardiovascular responses induced by enhanced baroreceptors and chemoreceptors activity during repetitive exercise sessions. In previous studies we have already shown in trained normotensive and spontaneously hypertensive rats that baroreceptors’ and chemoreceptors’ inputs are responsible for cardiovascular adjustments induced by exercise training (Cavalleri et al., 2011; Cruz et al., 2013) and that improvement of cardiovascular control was blocked by SAD (Ceroni et al., 2009). What we prove now is that sinoaortic denervation is also able to drive neuroplastic changes within supramedullary autonomic circuitry. Altogether these studies indicated that afferent inputs originated from arterial baroreceptors and chemoreceptors are the main stimuli to drive both activity-induced (increased signaling during repetitive exercise sessions) and inactivity-induced plasticity (in the absence of peripheral afferent signaling) of central autonomic pathways and the functional responses associated with them. It should be noted that afferent signaling changes should not be the only mechanism to drive plastic changes in autonomic circuitry. Bornemann et al. (2019) recently showed that a long-lasting mental training intervention influenced the parasympathetic modulation of heart rate variability in individuals carrying the homozygous allele AA of the oxytocin receptor gene. It remains to be determined whether this or other stimuli do change the plasticity of preautonomic OTergic pathways.

An important finding of the present study was that SAD blocked training-induced augmentation of OTergic projections directed not only to DMV and RVLM (the origin of preganglionic parasympathetic and premotor sympathetic neurons projecting to heart and vessels, respectively), but also those projecting to NTS and CVLM nuclei, the exact location of NORergic neurons that send afferent information to the PVN OTergic neurons. This result reinforces previous observation by Peters et al. (2008) that OT released from PVN descending projections acts on a subset of second-order neurons within the NTS to increase the release probability of glutamate and facilitate neuronal depolarization, thus enhancing synaptic transmission of afferent signals conveyed by peripheral receptors. In addition, it discloses that PVN modulation of afferent signaling also occurs within the CVLM, the other source of ascending NORergic information and that baroreceptors’ and chemoreceptors’ ablation largely reduces the hypothalamic modulation of other afferent signals such as those carried by cardiopulmonary and skeletal muscle receptors.

To investigate whether training and SAD effects were or not specific to preautonomic OT neurons we also analyzed their effects on the density of DBH+ fibers and OT+ neurons within the magnocellular PVN where neuroendocrine OT neurons are located. SAD still reduced ascending noradrenergic fibers and the expression of magnocellular OT+ neurons, suggesting that afferent information conveyed by baroreceptors and chemoreceptors are important to maintain the tonicity of these neuroendocrine neurons. Indeed, it is known that baroreceptors’ activity modulates plasma levels of oxytocin in response to changes in blood volume and/or osmolality (Sladek et al., 2015). On the other hand, exercise training did not change expression of local DBH+ fibers and OT+ neurons, indicating that exercise-induced neuronal plasticity was specific for the autonomic modulatory circuitry. Consistent with these observations previous studies showed that exercise training decreased the input/output function of magnocellular PVN neurons (Jackson et al., 2005) and reduced plasma OT levels in exercise trained rats (Braga et al., 2000).

Notice that, in opposition to drastic neuroplastic and functional effects caused by SAD, DBH-saporin treatment induced a partial blockade of noradrenergic transmission within the hypothalamus, reduced the density of OT+ neurons within PVN preautonomic nuclei and caused a mild reduction in OTergic terminals into the NTS, but did not interfere with training-induced increase of OTergic immunoreactivity within NTS and DMV, nor blocked the improvement in the reflex control of the circulation. These observations indicate that, whether baroreceptors and chemoreceptors are active and part of afferent ascending information to the hypothalamus is still intact, the beneficial effects of training are present, reinforcing the essential role of peripheral receptors in driving neuronal plasticity and the consequent functional responses.

## Limitations

One limitation of our study is that we did not identify by retrograde labeling the PVN preautonomic neurons projecting to NTS, DMV, CVLM, and RVLM nuclei. For this reason, care was taken to microinject anti-DβH-saporin and to analyze OT+ neurons within specific brain coordinates for the ventromedial and the posterior PVN nuclei, two well-known autonomic areas modulating cardiovascular control.

## Conclusion

In conclusion this study demonstrated that the inputs conveyed by baroreceptor and chemoreceptor afferents during daily exercise sessions are essential to drive training-induced plasticity within brainstem-hypothalamic neuronal circuitry involved in the autonomic control of the circulation and the concurrent beneficial cardiovascular adjustments Our data also showed that chronic absence of afferent peripheral signaling is accompanied by marked reductions in the density of ascending noradrenergic inputs from brainstem to PVN and in the immunoreactivity of OTergic preautonomic neurons projecting from the PVN to the brainstem, thus blocking training-induced neuroplasticity and functional adjustments in exercised sinoaortic denervated rats. Therefore, our data indicated that afferent inputs conveyed by arterial baroreceptors and chemoreceptors are the main stimuli to drive both inactivity-induced and activity-dependent neuroplasticity within the autonomic circuitry.

## Data Availability Statement

All datasets generated for this study are included in the article/Supplementary Files.

## Ethics Statement

The animal study was reviewed and approved by Institutional Animal Care and Use Committee of the University of São Paulo, in compliance with the Ethical Principles in Animal Research of the Brazilian College of Animal Experimentation.

## Author Contributions

CR-S and DB participated in the research design, performed the experiments, analyzed, and interpreted the data. AC performed the experiments and analyzed the data. CR-S wrote a draft of the manuscript. LM conceived, designed and coordinated the research, wrote and revised the manuscript. All authors read and approved the final draft of the manuscript.

## Conflict of Interest

The authors declare that the research was conducted in the absence of any commercial or financial relationships that could be construed as a potential conflict of interest.

## Supplementary Material

FIGURE S1 Video showing the tridimensional reconstruction of an OT+ neuron (red) taken from the PVN*post* of a SHAM-S rat showing DBH+ terminals co-localized with neuronal cell body and proximal dendrites (contacting boutons in blue) and DBH+ projections (green) that pass through the PVN but did not contact this specific OT+ neuron.

FIGURE S2 Video showing the tridimensional reconstruction of an OT+ neuron (red) taken from the PVN*post* of a SHAM-T rat showing DBH+ terminals co-localized with neuronal cell body and proximal dendrites (contacting boutons in blue) and DBH+ projections (green) that pass through the PVN but did not contact this specific OT+ neuron.

FIGURE S3 Video showing the tridimensional reconstruction of an OT+ neuron (red) taken from the PVN*post* of a SAD-S rat showing DBH+ terminals co-localized with neuronal cell body and proximal dendrites (contacting boutons in blue) and DBH+ projections (green) that pass through the PVN but did not contact this specific OT+ neuron.

FIGURE S4 Video showing the tridimensional reconstruction of an OT+ neuron (red) taken from the PVN*post* of a SAD-T rat showing DBH+ terminals co-localized with neuronal cell body and proximal dendrites (contacting boutons in blue) and DBH+ projections (green) that pass through the PVN but did not contact this specific OT+ neuron.
